# Supplementary material for: Trading patients’ choice in providers for quality of maternity care? A discrete choice experiment amongst pregnant women
Source: PLoS One. 2020 Apr 24;15(4):e0232098. doi: 10.1371/journal.pone.0232098 (PMC7182251; doi:10.1371/journal.pone.0232098)
Supplement: S1 File — (DOCX) [file pone.0232098.s001.docx]

**Supporting File S1: Background of Dutch health care system and bundled payment model for maternity** **care**

***S1.1 The Dutch Health Care system***

The Netherlands has a complex health care system. Insight in the structure of the system may help to appreciate the significance of the introduction of the bundled payment model and the results of the present study. This Supplementary information is mainly based on (29, 33-35).

**Health system reforms**

Since 2006 market forces and competition are increasingly introduced, aiming to keep health care affordable while ensuring good quality care and accessibility for all. The health care market is not an entirely free market; it is a regulated market, as it is subjected to laws and regulation to safeguard public interest. Quasi-governmental, independent oversight bodies monitor whether these rules are observed by the market players (35). To make the health care market work, the stakeholders in health care, i.e. the care consumers, the providers of care and the health insurers, were assigned a much more prominent role, while the government, although still pulling the final strings, assumed a less controlling role. The main features of the new health system are a mandatory ‘basic’ health insurance for everybody that covers essential medical care, mandatory acceptance for the ‘basic’ health insurance by the insurer, enrolees are allowed to switch insurer at the beginning of each year, there are in-kind and restitution policies and there is risk equalization for insurers. Health insurers are private companies, although not all of them are profit-driven, price negotiations are limited, and there is selective contracting on a limited number of conditions. Traditionally, the Dutch health care system can be characterized as primary care oriented (35). Almost every Dutch citizen is assigned to a single general practitioner (GP). The GP is the first point of contact with the health care system and acts as gatekeeper for secondary care. Specialist care is only accessible with a referral of the GP. Dutch primary care is also highly accessible.

**Health care markets**

The health system reforms introduced market forces into the health care market to a far wider extent than before. The three market players, i.e. the patients or consumers, the care providers and the insurance companies, were consigned far more prominent roles in making the health care market work. The health care market consists of three subsidiary markets: the health care provision market, the health care purchasing market and the health insurance market. The three markets are interrelated.

On the health care purchasing market health insurers purchase care from health care providers. For this market to work properly, they should purchase good-quality care at competitive prices. Insurers indicated, however, that quality of care played hardly any role in the purchase of care, because of a lack of relevant information (5). Extensive efforts have been made to make quality of care more transparent. Results, although still limited, are made accessible through websites.

Slowly but gradually, health insurers are starting to purchase care on the basis of quality. The four main health insurance insurers ( Zilveren Kruis Achmea, VGZ, CZ and Menzis), together about 14,6 million insured in 2018 of the about in total 17 million Dutch citizens) (36) have formulated quality standards for a number of surgical procedures. Hospitals that fail to meet these standards will not be contracted and/or will not get a favourable rating. Selective contracting by health insurers is still limited, but it is expected to grow considerably in the near future. Competition on price is possible to a certain extent depending on the type of care. The influence of health insurers on the purchasing market has probably been most pronounced in relation to medicines, due to the introduction of the Medicine Reimbursement System (Geneesmiddelen Vergoedings Systeem (GVS)). This system presents a ‘preferred’ medication list; unless medically indicated, only preferred medicines are reimbursed by the health insurer.

On the health insurance market health insurers supply health insurance, which is purchased by consumers. Since the Zvw, all health insurers are private companies and are allowed to make a profit and pay dividends to shareholders (35). However, there are a number of health insurance companies that operate on a non-profit basis. Health insurers are allowed to compete on quality of care, services and premium. After the introduction of the Zvw and the mandatory basic health insurance in 2006, competition among health insurers has been especially fierce on premium, even to the extent that they incurred losses. They made a profit on the basic insurance for the first time in 2009. Competition on coverage of the basic health insurance package is hardly possible, as under the Zvw coverage is the same for all basic packages. It is allowed for insurers to offer a slightly extended basic package to their enrolees, but this is by no means common practice. For the insurance market to work, consumers need to be able to switch health insurers. This is provided for by the Zvw, which allows the insured to change insurer at the beginning of each year. In 2006, 18.1% of the enrolees took advantage of this provision and switched. Since then, this percentage dropped to pre-Zvw levels of about 3.5% to steadily rise again and stabilize around 6,2% for 2018 (36). To compensate insurers for enrolees with predictably higher care consumption and thereby to prevent risk selection, there is a comprehensive risk equalization scheme. The scheme distributes funds from across the health insurers on the basis of the risk-profiles of enrolees.

On the health provision market, health care suppliers provide care to care consumers. Still, as previously stated, information on quality of care is hardly available, making it hard for the care consumer to make an informed choice regarding care providers. Consumers are increasingly using the internet to look for information on care providers and quality of care. For a large number of care providers (some) quality data are still lacking.

***S1.2 Key premises of the Dutch bundled payment model for materinity care***

Since 2017, it is possible for health insurers and maternity care providers to engage in bundled payment for maternity care. From 2017 to 2022, bundled payment contracts are possible for small-scale projects and on an experimental basis. Under the bundled payment system, insurers pay a single fee (depending on a combination of modules, see below) to a principal contracting entity — the integrated maternity care organisation (imco) — to cover a full range of maternity care services for a pregnancy. An imco is a newly created legal entity in the health care system, formed by multiple care providers, who are often community midwife practices, gynaecologists and postpartum maternity care organisations (In Dutch: ‘kraamzorg’). By signing the BP contract, the imco assumes both clinical and financial responsibility for all care delivered within the bundle to women receiving all maternity care within the organisations participating in the imco. When any maternity care is received outside of the IMCO the ‘bundle breaks’ and all services are claimed by the separate care providers via the former fee for service system. For the various components of maternity care, the imco either delivers services itself or subcontracts other care providers. The term ‘integrated maternity care organisation’ denotes the legal entity that is the prime contractor in a bundled payment contract; it does not refer to the team of health care providers that deliver the actual services. However, so far, all maternity care services are typically delivered by providers directly participating in the imco.

The bundled payments supersedes traditional health care purchasing market into two segments — one in which insurers contract care from IMCO’s and one in which IMCO’s contract services from individual providers, (see Figure S1.1). On both markets, fees are freely negotiated. General decisions about services to be covered in the maternity care bundle were determined at a national level and are codified in the Health Care standard ‘Integrated birth care’ (37), which was approved by all national provider and patient associations. Except for two, all services in the maternity care bundle are provided free of charge, since they are covered by the standard ‘basic’ insurance package mandatory to all Dutch citizens. The two exceptions are out of pockets payments for a part of in hospital delivery without medical indication and a part of post partum maternity care. Post partum maternity care is delivered at home and includes care for mother and child, (preventive) screening and guidance in setting up the new family. All maternity care services and activities necessary to ensure cooperation and coordination between the health care providers are covered in the bundled payment contract. In addition, imco’s are provided with an additional payment during the first 2 to 3 years to aid in setting up appropriate governance, administrative and financial structures. Health insurers negotiate a single contract with each imco to cover the entire set of agreed services for one episode of maternity care.

***Figure S1.1: Outline of the Bundled Payment model***

**Bundled payment contract consists of nine modules**

A bundle for maternity care, encompassing all maternity care for a pregnancy delivered by an imco as chosen by the client, consists of nine modules. When a client opts to receive any maternity care outside of the imco’s services, the separate care providers claim all services separately via the former fee for service system. For each module, insurer and imco freely negotiate a fee. The care allotted to a certain module is determined by the independent quasi-governmental oversight body Health Care Authority (in Dutch: Nederlandse Zorgautoritei). As depicted in Figure S1.2, the modules are categorised by prenatal, natal and postnatal phases. When a pregnancy terminates before 16 weeks, a single separate module is claimed. For all longer pregnancies, one module per phase is claimed plus the post partum maternity care hours. Within each phase, a division between regular and complex care is exists. For deliveries in hospital without a medical indication a separate module is created to accustom the associated out of pocket payment. Finally, post partum maternity care is claimed per hour. The maternity care bundle for a successful pregnancy consist of four modules; one from the prenatal phase, the natal phase, the postnatal phase and post partum maternity care.

***Figure S1.2: Scheme for the 9 modules in the Dutch maternity care bundle***
