# Supplementary material for: Trading patients’ choice in providers for quality of maternity care? A discrete choice experiment amongst pregnant women
Source: PLoS One. 2020 Apr 24;15(4):e0232098. doi: 10.1371/journal.pone.0232098 (PMC7182251; doi:10.1371/journal.pone.0232098)
Supplement: S2 File — (DOCX) [file pone.0232098.s002.docx]

**Supplementary file S2 Long list potential attributes**

**Table S2.1 Long list attributes, based on literature scan**

| **Attribute** | **Source** |
| --- | --- |
| Individualised care | (8) |
|  |  |
| Involvement in decision-making | (8-10) |
| Information provided by health care professionals | (11, 12) |
| Emotional support by health care professionals | (12) |
| Working in partnership with the midwife | (8) |
| Conflict during negotiation of the birth plan | (38) |
| Continuity of midwife | (13) |
| Responsibility | (11) |
|  |  |
| Type of birth setting | (9, 10, 14) |
| Place of giving birth | (10, 14, 15, 17) |
| ‘Homely’ environment/atmosphere | (13, 39) |
| Availability of competent health care professionals | (12) |
| Avoidance of medical interventions | (15) |
| Avoidance of early postnatal transfer | (15) |
| The local maternity facilities | (11, 15) |
| Opportunities to rest/recovery | (11) |
| Facilities provided for partner/family | (11) |
| Content and number of home visits | (17, 18) |
| Confidence in the staff | (15) |
|  |  |
| Process of delivery | (40) |
| Possibility of pain relief (including availability of a birthing pool) | (8-11, 13, 14, 41-43) |
| Mobility during labour (confined to bed on an intravenous drip or able to move around) | (42) |
| Mode of delivery | (42) |
|  |  |
| Autonomy | (11, 38, 44-46) |
| Confidence in the normalcy of birth, body’s ability to give birth, and birth attendant | (39) |
| Moral, physical, and emotional ‘fitness’ for pregnancy | (11) |
|  |  |
| (feelings of) safety | (11, 12, 15, 16, 39, 44, 45) |
| The mother’s concerns about the child’s condition | (12) |
|  |  |
| Distance, time travelled to delivery unit | (13) |
| Transport during birth in case of complications (to a different place) | (14) |
| Ease of access | (15) |
|  |  |
| Previous birth experiences | (15, 47) |
| The beliefs and values of the partner, family and friends | (16) |
| Reputation maternity care | (15, 16) |
|  |  |
| Maternal health ante partum | (40) |
| Maternal outcome | (40) |
| Neonatal outcome | (40) |
|  |  |
| Co-payment for childbirth | (14) |
| Lack of insurance | (39) |
| Access to private or publicly funded models of care | (47) |
|  |  |
| Number of appointments | (17, 18) |
| Number of visits to the hospital with perceived labor pains before actual admission to the labor ward | (42) |
|  |  |
| Telephone advice line provided | (17) |
|  |  |
| Hours of home-help provided after birth | (18) |
|  |  |
| Antenatal classes | (48) |
|  |  |
| Internet | (48) |
|  |  |
| Religious reasons | (39, 47) |
| Cultural and historical associations of birth and safety | (11, 47) |
| Country of origin | (47) |
