# Supplementary material for: Trading patients’ choice in providers for quality of maternity care? A discrete choice experiment amongst pregnant women
Source: PLoS One. 2020 Apr 24;15(4):e0232098. doi: 10.1371/journal.pone.0232098 (PMC7182251; doi:10.1371/journal.pone.0232098)
Supplement: S3 File — (DOCX) [file pone.0232098.s003.docx]

**Supplementary file S3: Introductory text to DCE questionnaire**

Dear expecting mother,

The current study deals with maternity care. This entails care services prior to, during, and after the birth of a child. Increasingly, maternity care is organised in networks in which midwifes, hospitals and maternity care organisations cooperate.

[…]

**What do we ask you to do?**

We ask you to remember the moment that you found out that you were pregnant and needed to decide which maternity care provider to choose to guide you during pregnancy and delivery. We will ask you a number of times to choose from two options.

Below you will find information of the choices we will ask you to make, The following aspects will be covered.

**Information between care professionals**

The maternity care providers can exchange patient information in different ways. In this study you will find the following situations:

- All your maternity care providers work from a single patient file and are well informed about your status.
- Your maternity care providers exchange patient information by e-mail telephone and fax, increasing the chance that they are not always up to date about your status.

**Information to you by the midwife**

The midwife informs pregnant women about pregnancy and delivery. This can take place in different forms. In this study you will find the following forms:

***Information pregnancy and delivery, ask questions, phone, email, text***

- *The midwife informs on pregnancy and delivery such as blood tests and ultrasounds, performs check-ups and answers all questions. Later contact is always possible by phone, email or texting.*

***Information pregnancy and delivery, ask questions, folders. websites, later more questions***

- *The midwife informs on pregnancy and delivery such as blood tests and ultrasounds, performs check-ups and answers questions during consultation. For further information the midwife refers to folders and websites. In a later consultation you can ask more questions*.

***Information pregnancy and delivery, ask questions, folders***

- *The midwife informs on pregnancy and delivery such as blood tests and ultrasounds, performs check-ups. For questions, the midwife refers to folders and websites.*

**Information by friends and family**

- You hear positive stories about the maternity care providers from your familiy and friends.
- You hear both positive and negative stories about the maternity care providers from your familiy and friends.

**Organisation of maternity care**

There are different ways to organise maternity care . In this study you will find the following forms:

***All care in one organisation. You cannot choose outside this organisation***

- You can enrol in a number of midwife organisations that cooperate with one single hospital land one organisation for postnatal care. You can only turn to these midwifes, and you cannot deliver your baby in another hospital, and postnatal care can only be delivered by this postnatal care organisation.

***All care in one organisation. And an option to choose a provider outside the maternity care organization.***

- You can enrol in a number of midwifery organisations that cooperate with one single hospital land one organisation for postnatal care. On your own initiatieve you may switch to a midwife from another practice, deliver your baby in another hospital and arrange for other postnatal care.

***All care arranged for separately***

- You enrol separately in a midwifery organisation, a hospital and a postnatal care organisation, all to your own preferences.

**Travel distance**

- The midwife holds practice 5 minutes by bike from your home; hospital is 5 minutes by car
- The midwife holds practice 10 minutes by bike from your home; hospital is 10 minutes by car
- The midwife holds practice 15 minutes by bike from your home; hospital is 15 minutes by car
- The midwife holds practice 20 minutes by bike from your home; hospital is 20 minutes by car

**Pain Relief**

From previous studies we know that availability of pain relief (or anaesthetics) are important to women during labour. However, availability of pain relief depends not so much on the maternity care organisation, but on place of delivery. We therefore ask you to assume that pain relief will be available conform to your preferences.
