# Supplementary material for: Trading patients’ choice in providers for quality of maternity care? A discrete choice experiment amongst pregnant women
Source: PLoS One. 2020 Apr 24;15(4):e0232098. doi: 10.1371/journal.pone.0232098 (PMC7182251; doi:10.1371/journal.pone.0232098)
Supplement: S5 File — (DOCX) [file pone.0232098.s005.docx]

S5 Codebook to dataset S4

| Variable name | Variable label | values | Values lables |
| --- | --- | --- | --- |
| RESP_ID | Id of respondent (anonimized) | 1-611 |  |
| VERSIE | Identifier of block in the design | 1-3 |  |
| set | Identifier of cenario in questionnaire | 1-36 |  |
| scenario | Identifier of scenario in choice set | 1-3 |  |
| aantal | Number of choices in choice set | 3 |  |
| choice | Answer respondent: chosen scenario | 0-1 | 0=not chosen; 1=chosen |
| epd | Variable: Information exchange between health care providers by EMR or not | -1, 1 | -1= by phone, mail fax etc; 1= by EMR |
| info | Variable: Information from midwife | 1-3 | 1= all information at intake orally; 2= part orally, part leaflets; 3= orally limited, rest infor leaflets and later stage |
| infomax | Variable maximum info | -1,0,1 | 1= (info =1)  0=(info=2)  -1 = (info=3) |
| infomed | Variable medium info | -1, 0,1 | 1= (info = 2)  0 = (info =1)  -1 = (info= 3) |
| soc | Var: information from family | -1, 1 | -1= family both positive and negative; 1 = family is positive |
| igo | Organization of maternity care | 1-3 | 1= all orgs separately; 2=1 org, option one provider external; 3 = one organization, no choice. |
| igoecht | Var Maternity care org part fixed, part choice | -1, 1 | 1= (igo=2)  0 = (igo=1)  -1 = (igo=3) |
| igovrij |  | -1, 1 | 1 = (igo=3)  0 = (igo=2  -1 = (igo=1) |
| cost | Travel time | 0.5; 1;1.5;2 | 0.5=5 minutes  1=10 minutes  1.5=15 minutes  2=20 minutes |
| VR01 | Educational level | 1-8 | 1= lowest  7= highest  8=open/else |
| LEEFTIJD_MOEDER | Age mother | 19-45 | years |
| eerstkind | First child? | 0-1 | 0= no; 1=yes |
| urbgr | Urbanisation level | 1-5 | 1= very strongly urbanized  2= strongly urbanized  3=average  4= little  5= not urbanized |
