# Supplementary material for: Trading patients’ choice in providers for quality of maternity care? A discrete choice experiment amongst pregnant women
Source: PLoS One. 2020 Apr 24;15(4):e0232098. doi: 10.1371/journal.pone.0232098 (PMC7182251; doi:10.1371/journal.pone.0232098)
Supplement: Attachment — Submitted filename: response_to_reviewers_R2_20200221_subm.docx [file pone.0232098.s007.docx]

Reviewers' comments:

Reviewer's Responses to Questions

**Comments to the Author**

1. If the authors have adequately addressed your comments raised in a previous round of review and you feel that this manuscript is now acceptable for publication, you may indicate that here to bypass the “Comments to the Author” section, enter your conflict of interest statement in the “Confidential to Editor” section, and submit your "Accept" recommendation.

Reviewer #1: All comments have been addressed

Reviewer #2: (No Response)

2. Is the manuscript technically sound, and do the data support the conclusions?

The manuscript must describe a technically sound piece of scientific research with data that supports the conclusions. Experiments must have been conducted rigorously, with appropriate controls, replication, and sample sizes. The conclusions must be drawn appropriately based on the data presented.

Reviewer #1: Yes

Reviewer #2: Partly

3. Has the statistical analysis been performed appropriately and rigorously?

Reviewer #1: Yes

Reviewer #2: Yes

4. Have the authors made all data underlying the findings in their manuscript fully available?

The [PLOS Data policy](http://www.plosone.org/static/policies.action#sharing) requires authors to make all data underlying the findings described in their manuscript fully available without restriction, with rare exception (please refer to the Data Availability Statement in the manuscript PDF file). The data should be provided as part of the manuscript or its supporting information, or deposited to a public repository. For example, in addition to summary statistics, the data points behind means, medians and variance measures should be available. If there are restrictions on publicly sharing data—e.g. participant privacy or use of data from a third party—those must be specified.

Reviewer #1: Yes

Reviewer #2: Yes

5. Is the manuscript presented in an intelligible fashion and written in standard English?

PLOS ONE does not copyedit accepted manuscripts, so the language in submitted articles must be clear, correct, and unambiguous. Any typographical or grammatical errors should be corrected at revision, so please note any specific errors here.

Reviewer #1: Yes

Reviewer #2: Yes

6. Review Comments to the Author

Please use the space provided to explain your answers to the questions above. You may also include additional comments for the author, including concerns about dual publication, research ethics, or publication ethics. (Please upload your review as an attachment if it exceeds 20,000 characters)

**Comment reviewer 1:**
Reviewer #1: Thank you to Lambooij and co-authors for the revised version of their manuscript. Following their effort, the quality and clarity of the article have improved considerably.

I have few additional minor remarks that I feel should still be addressed before publication.

**Authors’ response**

We thank the reviewer for the compliments and critical and constructive assessment of the paper at this current round. In the following we address the comments that were addressed point by point.

**Comment reviewer 1**:
General comment:
Thought the form is significantly better (with the exception of the discussion which seems of lesser quality), I would suggest an additional review to check for punctuation, spacing between words and words/punctuation, and for form (eg: Bundled Payment, with capitals or not? be consistent).

Specific comments (refer to the clean version of the manuscript, not the track/trace) (words in capital letters are my suggestions for addition):

Abstract:
-if possible (if words allow) I would contextualize the background to the Netherlands. Eg: ‘…describes a DUTCH study…’
-third line ‘….pregnant women’s….’ NOT ‘…[THE] pregnant women’s…’

**Authors’ response**

Thank you. We added ‘Dutch’ and deleted ‘the’ in the abstract. Furthermore we checked the consistent description of ‘Bundled Payment’ and punctuation throughout the manuscript.

**Comment reviewer 1:**
Introduction:
-the second sentence of the second paragraph (starting with ‘Empirical…) is not grammatically correct

**Authors’ response**

Thank you. We changed the sentence into the following:

“Empirical associations have been found between use of integrated care and increased patient satisfaction, perceived quality of care and patient access to services”

-last sentence in the first page, I would specify to: ‘in THE six DUTCH regions…’

**Authors’ response**

We added ‘Dutch’

-second page of the introduction, second paragraph, sentence starting with ‘But also…’: is ‘birth place process’ the right term? Also, is reference #10 the correct one? I couldn’t find any info on birth place process in reference #10

**Authors’ response**

Thank you for your observation. It should be ‘and influence in the birth process’. We changed this accordingly. In response to this comment to find out what went wrong with the reference, we found that our literature software End Note had caused and error involving reference 10 and reference 3. They referred to the same paper but were included as different references. We corrected this and added the correct reference.

-last paragraph second page, ‘the results of this study are relevant for policymakers, as THEY PROVIDE’ and not it provides

**Authors’ response**

We changed this accordingly, thank you.

**Comment reviewer 1:**
Results:
-Sample section, last sentence: ‘…and women who were pregnant with their… --> should this be: ‘…and MORE women who were pregnant with their first child….'?

**Authors’ response**

Indeed, adding ‘more’ adds clarity to what we mean to say. We added ‘more’ to the sentence.

**Comment reviewer 1:**
Discussion:
-second sentence, I would suggest: ‘For SOME women the unlimited provider choice is the most important aspect, and THEY find this more….’

**Authors’ response**

That is a good suggestion. Thank you. We changed this accordingly.

-first sentence second paragraph, remove the A before about: ‘…that people had [a] about a one in four….’

**Authors’ response**

Thank you for your thorough reading of the paper. We changed this in line with the suggestion.

-first sentence second page: ‘….difficult-to-measure (add the dash between to and measure) aspects such as [of] harder-to-measure…’ AND try not to repeat ‘such as’ in the same sentence so closely

**Authors’ response**

We added the dash and rephrased the sentence, removing a “such as”:

“Future studies may yield relevant new information on both relative importance and possible interactions of difficult-to-measure aspects and harder-to-affect aspects such as women’s previous experiences compared to factors that can be affected by maternity care providers or policymakers.”

-last paragraph second page, first sentence: remove the last word ‘attributes’, you already mentioned it earlier in the sentence, that is enough

**Authors’ response**

We removed the last word ‘attributes’.

-last line second page: scenario’s is no plural in English (that is the Dutch form), in English it’s scenarios

**Authors’ response**

Thank you. We corrected this.

-third page, third line: ‘intrinsic factOR’

**Authors’ response**

We understand the suggestion of the reviewer. However, we mean to imply that trading is a fact of life when making choices. So even though ‘factor’ would fit in this sentence. We prefer to maintain the word ‘fact’.

-end of second paragraph: ‘Since DCEs’ main objectives ARE to describe…’ OR ‘Since DCEs’ main OBJECTIVE is to describe…’

**Authors’ response**

Thank you. We corrected this:

“Since DCEs’ main objectives are to describe scenarios as close to a real decision making situation as possible […]”

-third paragraph: third and fourth sentence start with ‘this means’…it feels you are trying to explain a concepts with several sentences without succeeding. Can you limit the use of ‘this means’ to once?

**Authors’ response**

We adapted this section:

“This means that in the current study, the respondents are likely to have more information on factors that affect the quality of maternity care than in real life. Consequently the relative importance of those factors may be overestimated compared to clearly visible factors such as travel time or advice from the midwife.”

-third page last sentence: ‘….in practice THE WOMEN...’ --> instead of ‘they’ I would write ‘the women’, since the subject in the first part of the sentence is the study, not the women.

**Authors’ response**

Thank you for pointing this out. We changed ‘they’ to ‘the women’.

-fourth page, twice used: ‘medical technical measures’ --> is this the right term? It was not used in the text before and it’s not an obvious term

**Authors’ response**

We mean to refer to ‘medical technical measures of quality’. We changed this to medical technical quality measures:

“In our case, we needed to exclude more medical technical quality measures (e.g. APGAR) because our main focus was to resemble a choice similar to a real-life choice as much as possible. When it comes to quality of care, it may be useful to compare the relative importance of medical technical quality measures of care and more patient- centred measures of quality of care in future studies.”

-fourth page, third line: I would remove ‘choice similar to a’

**Authors’ response**

Thank you for the suggestion. We removed the words accordingly.

-the very last sentence of the discussion seems to be speculation without references. It’s a peculiar way to end a discussion. I would revise it

**Authors’ response**

The statement indeed may have been phrased too strongly. We rephrased the sentence to refer more clearly to the results of the current study, making clear that the authors consider this to be a policy implication of the results.

“This study indicates that enabling provider choice under a bundled payment model is essential in order to develop delivery models that in meets the demands of all pregnant women”

**Comment reviewer 1:**
Conclusion:
-usually reference are not used in the conclusion, is references 14 needed there again?

**Authors’ response**

| We agree with the reviewer and deleted the sentence. |  |
| --- | --- |

**Comment reviewer 2:**
Reviewer #2: Authors have made an effort to address my comments. However, the following comments need additional attention.

**Comment reviewer 2:**
• Details about the variables, including coding, and the reason for their inclusion should be presented in the methods section. Supporting file S5 Codebook to dataset is fine but it is important to provide the above information in the methods section.

**Authors’ response**

We thank the reviewer for critically assessing the paper and constructive comments.

We agree with the reviewer that readers will need to be able to read more details in our methods section.

Therefore we added the following two blocks of text in the methods sections:

“This process led to the final set of attributes, levels and final rephrasing of attributes and levels (Table 1). The first three attributes are operationalisations of quality of care that can be observed by clients (indicated by a (Q) in de table), the final two attributes in table1 are operationalisations that affect the choice possibilities (C) of the women. The first attribute, “Information between care professionals by single EMR” is included to operationalize an essential part of integrating care services: access to relevant and up-to-date patient information by all care professionals [18]. Both the experts and the respondents in the pilots agreed to the relevance of this attribute. The second attribute, ‘Information to you by the midwife’ was included to operationalise the way the information from the health care organisation was given to the pregnant women. In order to make informed decisions, having access to sufficient and accurate information is essential. The third attribute ‘Advice by friends and family’ was an operationalisation of quality of care of the maternity care organisation. The Dutch National Health Care Institute (Zorginstituut Nederland) developed a set of quality indicators for maternity care. Following this set, in expert interviews and in the pilot test we tested the following options: (1) a standardized quality score on internet indicating whether the organisation was ranked in the top 20% or average, (2) percentage of complications of the maternity care organisation, presented on the internet, (3) a score on forced deliveries, presented as average, below average or above average, (4) average APGAR score of maternity care organisation, (5) reviews on the internet of experiences by other women. The experts preferred more objective measures (1-4), however, the respondents in our pilot study indicated that this information did not mean anything to them and that they would only value information related to quality of care presented to them by people that they knew. The fourth attribute ‘Organisation of maternity care’ was included to operationalize the consequences for the pregnant women of an integrated care organisation (IMCO). The final attribute ‘travel time’ was included as a consequence of integrating care; if maternity care organisations would integrate, this could imply that the nearest provider would not be cooperating with the IMCO that was chosen but women would need to travel for a longer time for their maternity care visits.”

And

“All variables were effect coded [27], except for travel time. This was recoded as follows: 5 minutes=0,5; 10 minutes=1; 15 minutes=1,5 and 20 minutes=2. The reference category for ‘info midwife med’ and ‘info midwife max’ was ‘info midwife minimum’ (level 1 in table 1). The reference category for ‘Org. partly fixed’ and ‘Org. no choice’ was ‘all care organized separately’ (level 1 in table 1).”

**Comment reviewer 2:**
• Authors should extend the discussion (the first 3 paragraphs) and add an interpretation of the results based on previous literature to establish convergent validity and to outline the implications for policy and research. I mean an interpretation of the findings on women’s preferences not the methodology aspects of the study. Why are these preferences observed? How do they compare to previous studies? What do they mean for policy and research?

**Authors’ response**

We agree with the reviewer that this is adds relevant information to the paper. We added paragraphs to the Discussion section, comparing our study to previous studies. We focus on a number of similarities and we mention a number of new insights, adding to previous work. We also added a paragraph discussing our view on the policy implications of the findings of our study.

Paragraphs in the discussion reflecting on previous research and our results:

“Our results are in line with previous findings that women are willing to trade between factors of maternity care [30]. What this study adds to the literature is the diversification in the population of interest: different women will make different choices when they can. This is in line with the previous finding that women differ in preferences related to service attributes [31]. A difference between that review and our findings is that the current study is restricted to attributes related to organisational aspects of maternity care services that are directly influenced by the design of the bundled payment model, while the underlying studies in the review also included other aspects (e.g. personal experiences, factors related more specifically to maternity care providers involved, such as continuity of midwife) [31]. We excluded personal experiences [32, 33] women’s beliefs and values [34] or type of birth setting [35-37], even though these factors are known to be relevant and to affect the preferences of pregnant women. The downside of restricting this study to factors that can be affected by the organisation of care is that we do not know the relative importance of those factors compared to the factors we did not include.

We also found that availability of pain relief was very important to women and partners [14, 31, 35-41]. Although pain relief is generally considered a highly important aspect of maternity care decisions, this is only very weakly linked to the organizations of integrated care and therefore not included in this study.

For the majority of the respondents we found that the information given by the midwife was weighed to be least important, and information given by friends and family was more important. This is in line with [42] who found that midwifes were not the main source of information for pregnant women, but that women wanted the option to discuss and consider their birth preferences throughout their pregnancy with multiple sources, not at a fixed point. This would mean that policymakers nor health care professionals are able to provide pregnant women with the type of information that they need in order to develop their preferences.”

Paragraph on policy implications:

“Current design of the Dutch bundled payment guarantees an unlimited choice for pregnant women via the existence of the bundle breaker. In practice, this means that when a pregnant women receives care from a care provider, which is not subcontracted by the IMCO, the bundled payment expires and all maternity care services are reimbursed via the existing payment model which is predominantly fee-for-services. This leads to an enormous administrative burden for care providers. In order to keep support among care providers for the bundled payment model the design of the bundle breaker preferably must be simplified in order to find a middle ground between unlimited provider choice and the administrative burden for care providers. Finding this optimum is not straight forward but is needed to keep support among both pregnant women and providers. The results of this study support current policy design of the bundle breaker, indicating that the current of the bundled payment with a bundle breaker appears to be close to the optimum of preferences of pregnant women.”

**Comment reviewer 2:**
• The selection bias and the limitations of it should be discussed more substantially to establish the possibility to extrapolate the results. Unless this is done, conclusions only refer to women in the register “The happy box” and not to all women in the Netherlands. The representativeness of the sample should be accounted for when making conclusions. In this regard, the validity of the current conclusions is unclear.

**Authors’ response**

The reviewer appears to consider the possibility that selection bias in the sample may be caused by the source we used to approach our respondents, affecting the results. We also considered this risk in advance and we therefore included control variables in the questionnaire to correct for possible biases. We selected the variables based on previous work and more or less ad hoc hypothesis of aspects that might affect preferences of pregnant women. We tested whether these sample characteristics affect the results, this turned out to be true for educational level, therefore we included educational level in the model. We consider the channel through which we contacted the hard-to-reach respondent group as instrumental, and tried to control for risks of bias in the analyses.

In the paper, the most important conclusions are drawn considering existing preference structures in the population. We presented our analyses to determine relevant class predictors “We tested the following parameters for a significant contribution to the class assignment model: education high/low, urbanisation level (5 categories), urbanisation city/rural, first child/gravidity status. ”

We found that educational level significantly affects preference structures, and the other aspects do not. We therefore included educational level as class predictor. We referred to evidence that higher educated respondents are likely to trade a larger number of attributes in their choices. We have no empirical indications nor hypotheses to predict directions of differences between people as to why having a first child or second/third/more, would affect the preferences of the women.

We therefore have no reason to assume that the known discrepancies between our sample and the general population would affect the four preference structures that we found. A different sample would likely give slightly different parameters, which may affect the relative importance of attributes within preference structures. However, given the sizes of most RIS’s it we find it unlikely that a another sample would yield shifts that are sufficiently large to thoroughly affect the ranking of the attributes.

Based on this, we have no reason to assume that in a sample with more lower educated women, more women who live in cities and more women with a multigravida status, different preference structures are likely to emerge.

Conclusions that we draw on the prevalence of the preference structures are based on the chance that each respondent has to belong to a class. Also here, a different sample is likely to yield slightly different parameters. However in the current sample the odds are divided evenly and the conclusions we draw take account of the uncertainty. Even though we cannot exclude that a different sample would yield different results, we see no reason why a different sample would lead to completely different results and affect the main conclusions to a large extent. We added these arguments to the text.

The section on the sample is now as follows:

“We found that in our sample, highly educated women, women from rural areas and women who were expecting their first child were overrepresented. Previous research has shown that higher educated respondents trade more of the included attributes than lower educated respondents in DCEs [42]. This could mean that in real life choices, a larger proportion of women weigh fewer of the maternity care aspects than the aspects that were included in the current study. Despite the overrepresentation of highly educated respondents, the sample benefits from the fact that all respondents had actual experience in the choice in the DCE because they were pregnant at the time of filling in the questionnaire. This meant that respondents were familiar with and had experience in the decisions they were asked to make in DCEs. In the analyses, we found that level of urbanisation, gravidity status did not predict the classes in the LCA. This implies that these person related factors do not affect the preferences structures that we found. If we would have had a sample with more highly educated women, living in a city it is likely that the parameters we would have found would differ. However, considering the relative importance of the attributes in the four preference structures, these differences in parameters would need to be very large to affect the ranking, and therefore the conclusions that we draw on the existence of the preference structures. We have therefore no reason to assume that the relative importance of the attributes in our results differ from the relative importance in real life choices, when just looking at the ranking of attributes. However the precise parameter values are likely to differ between the sample in this study and the general population [43]. We have therefore no reason to assume that a different sample would lead to very different conclusions concerning the preference structures of pregnant women in the Netherlands. ”

7. PLOS authors have the option to publish the peer review history of their article ([what does this mean?](https://journals.plos.org/plosone/s/editorial-and-peer-review-process#loc-peer-review-history)). If published, this will include your full peer review and any attached files.


If you choose “no”, your identity will remain anonymous but your review may still be made public.


**Do you want your identity to be public for this peer review?** For information about this choice, including consent withdrawal, please see our [Privacy Policy](https://www.plos.org/privacy-policy).

Reviewer #1: Yes: Elena Ambrosino

Reviewer #2: No
